# Effects of Post-Harvest Ozone Treatment on Some Molecular Stability Markers of *Amelanchier alnifolia* Nutt. Fruit during Cold Storage

**DOI:** 10.3390/ijms231911152

**Published:** 2022-09-22

**Authors:** Natalia Matłok, Tomasz Piechowiak, Miłosz Zardzewiały, Maciej Balawejder

**Affiliations:** 1 Department of Food and Agriculture Production Engineering, University of Rzeszow, St. Zelwerowicza 4, 35-601 Rzeszow, Poland; 2 Department of Chemistry and Food Toxicology, University of Rzeszow, St. Ćwiklińskiej 1a, 35-601 Rzeszow, Poland

**Keywords:** ozone, saskatoon berry, mechanical properties, microbial load, antioxidant potential, polyphenols

## Abstract

Fruits of *Amelanchier alnifolia* Nutt. ex M. Roem. (Nutt.) are a good source of bioactive compounds and vitamins. Due to the fact that the berries are a soft fruit, they require special procedures to increase their molecular and mechanical stability during cold storage. The study investigated the effects of ozone treatment applied cyclically (every 24 h) on selected chemical and mechanical parameters of saskatoon berries kept in storage. For this purpose, measurements were performed to assess changes in some molecular markers such as antioxidant potential, content of vitamin C, and total polyphenols, as well as microbial stress and maximum destructive force under uniaxial compression of samples. The effectiveness of the storage process was also assessed in relation to the conditions used by determining the proportion of fruit affected by diseases occurring in storage. The findings show that ozone treatment led to increased content of bioactive compounds at the initial stages of storage and resulted in decreased loss of water and bioactive compounds at the later stages. Ultimately, irrespective of the conditions applied during ozone treatment, it was observed that the growth of micro-organisms on the fruit surface was inhibited, and as a result, storage losses during the relevant period were significantly reduced.

## 1. Introduction

The saskatoon shrub (*Amelanchier alnifolia* Nutt.) is a plant in the family Rosaceae, native to southern Yukon and northwestern regions of North America [1]. Saskatoon berries are cultivated on a large scale in Canada [2]. The most popular cultivars of saskatoon berry include ‘Honeywood’, ‘Martin’, ‘Northline’, ‘Pembina’, ‘Regent’, ‘Smoky’ and ‘Thiessen’ [1,2,3]. In Europe, production of saskatoon berries is rather small, but it is constantly growing, mainly in Finland, the Czech Republic, Lithuania, Latvia, and Poland [4].

The content of nutritional and bioactive compounds in saskatoon berries depends on the maturity of the fruit, as well as growing and harvesting conditions, the genotype, and storage conditions [4,5]. Saskatoon berries have a low calories value (100 g of fruit provide approximately 85 kcal of energy); they are found with high vitamin content (tocopherols, riboflavin, ascorbic acid, pyridoxine, thiamine); they are an excellent source of minerals (manganese, magnesium, iron, calcium, potassium, and copper) [6], sugars (fructose, glucose, or sorbitol), organic acids, proteins, and pectins [2,4,7] and exhibit higher antioxidant activity than other fruits, such as strawberries or raspberries [6,8]. Polyphenols contained in saskatoon fruit exhibit strong antioxidant, anti-inflammatory, and antiallergic effects [5,9]. Saskatoon berries produce antineoplastic effects by preventing changes from taking place during oxidative stress and inflammation and by regulating carcinogenic and xenobiotic metabolising enzymes; various transcription and growth factors; inflammatory cytokines; and the subcellular signalling pathways of cancer cell proliferation, apoptosis, and tumour angiogenesis. Saskatoon berry consumption generates antidiabetic effects. A powder obtained from saskatoon berries containing cyanidin-3-galactoside and cyanidin-3-glucoside reduces glucose, insulin, and lipid levels in blood [8].

Following harvest, fruit retains the characteristics of living organisms, and as a result, metabolic processes continue to take place, leading to changes in the profile of the nutritional and non-nutritional compounds [10]. By applying various agents, it is possible to modify properties of fruit developing in storage. Standard procedures involving modification of the storage atmosphere and temperature make it possible to slow down the natural loss of bioactive compounds contained in fruit [11]. The scarce research reports related to this issue suggest that certain procedures can be applied to increase the contents of selected substances in the post-harvest period. One of these procedures involves regularly repeated ozone treatments applied to raw materials in storage [12,13]. Piechowiak et al. [14] observed positive changes in the contents of low-molecular-weight antioxidants in intercepted raspberries and blueberries. These changes were shown to be triggered by the activation of defence mechanisms against oxidative stress induced by gaseous ozone [14]. A modified version of this procedure was used to obtain fruits and selected plant parts with increased contents of selected bioactive compounds [12,15,16].

The purpose of this study was to identify the effects of ozone treatment on selected chemical parameters of saskatoon berries kept in storage. Measurements were performed to assess changes in antioxidant potential, contents of vitamin C, and total polyphenols, as well as mechanical properties and microbial stress. The effectiveness of the storage process was also assessed in relation to the conditions applied.

## 2. Results and Discussion

### 2.1. Water Content

Adverse changes that occur during the storage of raw plant materials, mainly soft fruits, include loss of turgor due to the decrease of water content [12].

**Figure 1:**
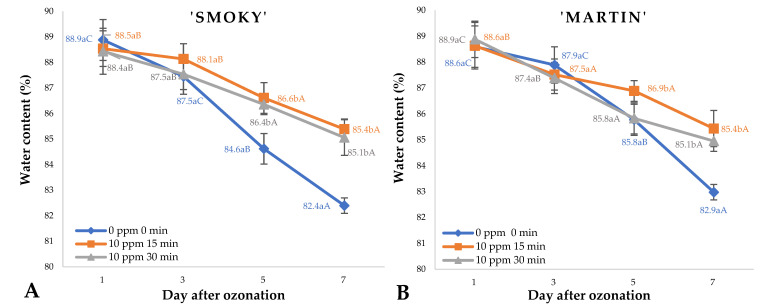
Water content and standard error of the means (%) in saskatoon berries of ‘Smoky’ (**A**) and ‘Martin’ (**B**) cultivars, relative to the dose of ozone (*n* = 3). Note: Differences in results between the dose of ozone on the specific days are indicated by different small letters, and differences between assessment days are indicated by different capital letters. Significance level at *p* < 0.05.

Measurement of water contents (Figure 1A,B) in saskatoon berries of ‘Smoky’ and ‘Martin’ cultivars, treated with ozone and untreated (control), showed a relationship between the ozone treatment conditions (concentration and time of exposure) and water loss during storage at 8 °C. There was a decrease in water content in fruit of *Amelanchier alnifolia* Nutt. kept in storage, irrespective of the variety or the conditions applied during the post-harvest treatment with ozone. The fruit samples kept in storage exhibited the characteristics of living organisms, which means that various life processes, including respiration and transpiration, continued to take place, leading to adverse changes in the quality of the stored raw material, mainly loss of water [12]. The largest loss of water in the samples was observed on day 7 of storage. However, this decrease was significantly related to the conditions applied during ozone treatment. Cyclical application of ozone treatment (ozone concentration of 10 ppm, exposure time 15 min per day) to saskatoon berries directly after harvest and during storage resulted in significantly decreased water loss compared to that observed in untreated fruit (control). The findings showed that water contents in ozone-treated samples of ‘Smoky’ and ‘Martin’ cultivars on day 7 of storage were 3.5% and 3.7% lower than in the samples examined directly after harvest. In the case of the untreated fruit samples, the decrease was twice as high, amounting to 6.5% (‘Smoky’ cultivar) and 6.2% (‘Martin’ cultivar). Similar correlations were identified on earlier days of storage, mainly on day 5 and when the other ozone doses were applied. The decrease in water loss observed in ozone-treated saskatoon berries was most likely associated with a reduced microbial load on the surface of the fruit, but this statement should be verified. Ozone, being a disinfectant, reduced microbial activity, preventing damage to the fruit surface, which led to reduced water loss and increased mechanical strength of the fruit [10]. It should be noted that by applying ozone treatment, it is possible to not only decrease water loss in stored fruit, but also to reduce financial damage resulting from weight loss and deteriorating quality of the stored material. The relationship between ozone treatment and decrease in water contents of plant material kept in storage has also been investigated by other researchers. Zardzewiały et al. [12] reported that rhubarb petioles subjected to treatment with gaseous ozone at a rate of 100 ppm for 30 min were found with water (weight) loss that was 13.11% lower, compared to the losses observed in untreated rhubarb samples. Likewise, Gorzelany et al reported beneficial effects of ozone treatment reflected by improved quality parameters and reduced loss of water content in cucumbers kept in storage [17].

### 2.2. Mechanical Properties

#### Compression of Independent Specimens

Important quality parameters of raw materials kept in storage include their mechanical resistance, which is directly related to their turgor value. These parameters mainly depend on both the rate of water loss, which is a natural phenomenon during storage of raw plant materials, and damage caused by the presence of microorganisms [17].

**Figure 2:**
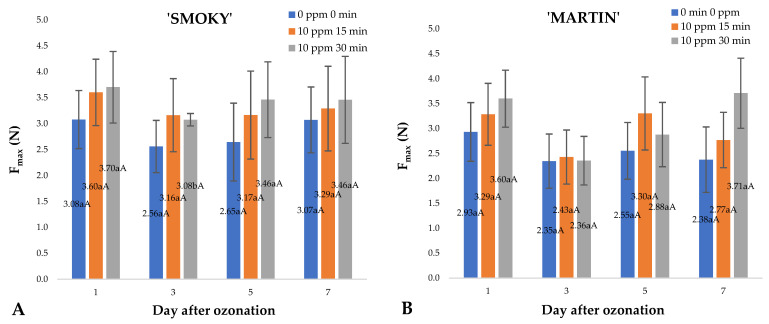
Mean values of maximum destructive force F_max_ and standard error of the means for saskatoon berries of ‘Smoky’ (**A**) and ‘Martin’ (**B**) cultivars, relative to the dose of ozone (*n* = 20). Note: Differences in results between the dose of ozone on the specific days are indicated by different small letters and difference between assessment days are indicated by different capital letters. Significance level *p* < 0.05.

In the case of saskatoon berries of the ‘Smoky’ cultivar, higher mean values of destructive force were observed (statistically insignificant) in the samples subjected to ozone treatment (Figure 2A,B). It was also observed that some increase in the destructive force (statistically insignificant) for the fruit corresponded to higher dose of gaseous ozone applied. However, the differences were not statistically significant. Analysis of this parameter is extremely important if ozonation process is applied during fruit storage. In the case of sea-buckthorn berries subjected to a single pre-storage ozone treatment, measurements showed a significant increase in the maximum destructive force for the sample in a uniaxial compression test. The differences were large enough to affect the overall assessment of the suitability of these fruits for consumption and processing [10]. However, in the case of the investigated raw material, the observed changes in the maximum destructive force, although not significant, confirmed the improved turgor value in the fruit, as shown in the analysis of changes in their water content (Figure 1A,B).

### 2.3. Microbial Load

The ozonation process makes it possible to significantly reduce the microbial load of raw plant materials. The micro-organisms contributing to fruit deterioration are largely accumulated on the fruit surface. Gaseous ozone primarily affects the surface, making it possible to remove such organisms to a large extent [18].

**Figure 3:**
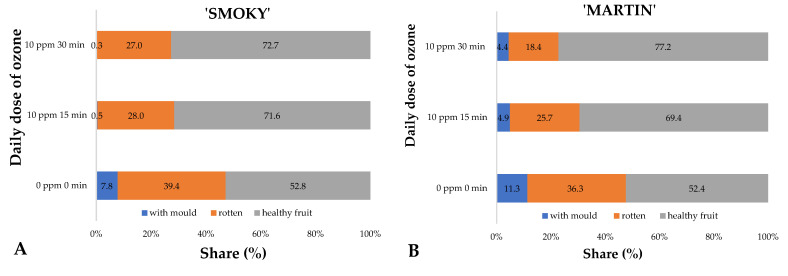
Mean rates of saskatoon berries of ‘Smoky’ (**A**) and ‘Martin’ (**B**) cultivars, infected with mould, rotting or healthy, in samples assessed on day 7 of the experiment, relative to the conditions of the ozone treatment applied.

In the case of saskatoon berries stored in an ozone atmosphere, a significant load of aerobic bacteria as well as yeast and mould were observed on the fruit (Table 1). Even only in the first day of storage, it was found that the ozone treatment positively affected the counts of these microorganisms. During fruit storage, the microflora abundance increased; however, the ozone treatment resulted in a decreased microflora growth rate compared to fruit in the control (untreated) sample. Uncontrolled growth of some of these micro-organisms results in visible lesions and damage to the fruit surface (Figure 3A,B), and consequently leads to infection with diseases occurring in storage that generate losses. The only effect related to reducing the microbial load was observed on day 1 of storage, in ‘Smoky’ fruit treated with ozone at a rate of 10 ppm 30 min^−1^; the treatment reduced the load by 0.74 log cfu (colony-forming unit), relative to the control sample. On the following days, the decrease in mould and fungal counts was less pronounced, yet microbial load values were lower in ozone-treated samples. Bacteria turned out to be more resistant to ozone. It is possible that these organisms had already penetrated the fruit and were thus protected from the direct effect of ozone. It should be noted that bacteria are mainly responsible for decay processes, which was confirmed by the results of the present study (Table 1). Similar correlations were identified in the case of blueberries stored in an atmosphere of gaseous ozone [19]. The ozone treatment applied to these fruits almost completely stopped grey mould growth, whereas anthracnose—found on the surface—developed from spores inside the fruit.

Possible effects of regularly repeated ozone treatment (every 24 h) on the shelf life of saskatoon berries were assessed on the final day of the experiment. To this end, the percentages of rotten, mould-infested fruit and fruit with no visible lesions or damage were identified in each variant examined [Figure 3A,B]. A significant relationship was observed between increasing doses of gaseous ozone and reduced losses occurring during storage in the fruit of both ‘Smoky’ (Figure 3A) and ‘Martin’ (Figure 3B) cultivars. In the case of the ‘Smoky’ cultivar, ozone treatment virtually completely inhibited the growth of vegetative forms of mould (thallus), the presence of which makes the fruit unsuitable for consumption. Notably, some fruits were found with early signs of the rotting process. Nevertheless, their proportion in the sample on average was about 10% lower than in the case of fruit which were not treated with ozone (control) (Figure 3A). Similar effects were observed in the case of saskatoon berries of the ‘Martin’ cultivar. The latter cultivar, however, was more susceptible to moulds, the incidence of which was significantly reduced by the application of gaseous ozone (Figure 3B). Analysis of the findings clearly shows that the ozone treatment applied to saskatoon berries kept in storage makes it possible to reduce losses, which directly affects economic gains. The most favourable effects, with losses reduced by ~20%, were observed in samples exposed to the highest doses of ozone.

### 2.4. Bioactive Compounds

#### 2.4.1. Total Polyphenolic Content

Gaseous ozone is an abiotic elicitor of defence responses in plant materials and fruit exhibiting properties of living organisms. As a result, a number of biochemical processes take place in the raw materials, leading to the formation of many secondary metabolites, mainly polyphenols and vitamins [14].

**Figure 4:**
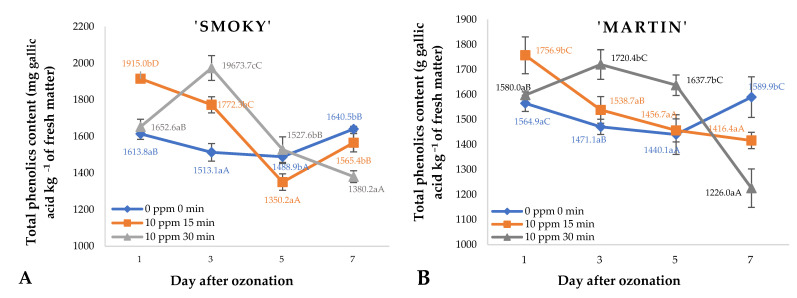
Total polyphenolic content and standard error of the means in saskatoon berries of ‘Smoky’ (**A**) and ‘Martin’ (**B**) cultivars, relative to the dose of ozone (*n* = 3). Note: Differences in results between the dose of ozone on the specific days are indicated by different small letters, and differences between assessment days are indicated by different capital letters. Significance level *p* < 0.05.

Ozone treatment applied immediately after harvest and cyclically during storage of saskatoon berries led to a significant increase in the total polyphenol contents of the raw material (Figure 4). The relatively best results in the case of the ‘Smoky’ cultivar were observed up to day 5 of storage in samples treated with gaseous ozone at a rate of 10 ppm 30 min. Only on day 7 of storage, fruit of this cultivar subjected to the latter treatment were found with a significantly lower polyphenol content than saskatoon berries from the other experimental variants. Likewise, the best effects of ozone treatment applied to saskatoon berries of ‘Martin’ cultivar during storage (Figure 4B) were observed in the samples treated with ozone at a rate of 10 ppm 30 min.

Changes in the total polyphenol content during storage are observed in all fruit species. As a rule, there is a decrease in polyphenol content, and its magnitude depends on the enhancement methods applied during storage. By applying enhancement involving ozonation, it is possible to stop this decrease to a large extent [20], and in some cases, a significant increase has been observed in the amount of these compounds in fruit kept in storage. Enzymes involved in the production of these secondary metabolites in fruit include L-phenylalanine ammonia lyase (PAL) [21], which is responsible for the transformation of these compounds into the mechanism that induces plant defence against stressors.

#### 2.4.2. Antioxidant Activities

**Figure 5:**
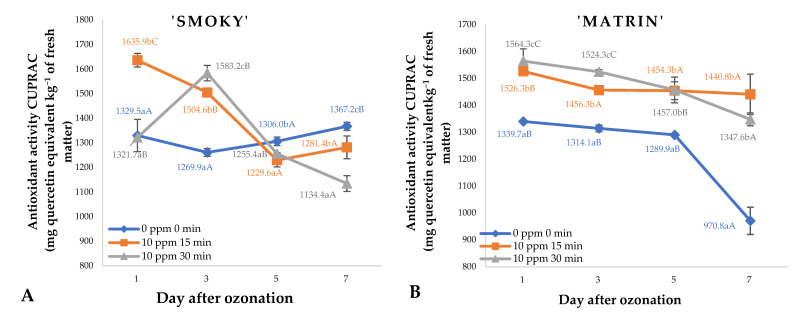
Antioxidant activity CUPRAC test and standard error of the means in saskatoon berries of ‘Smoky’ (**A**) and ‘Martin’ (**B**) cultivars, relative to the dose of ozone (*n* = 3). Note: Differences in results between the dose of ozone on the specific days are indicated by different small letters and differences between assessment days are indicated by different capital letters. Significance level *p* < 0.05.

Ozone treatment cyclically applied to saskatoon berries kept in storage affected the antioxidant potential of the raw material (Figure 5). The effect differed depending on the saskatoon berry cultivar and the duration of storage. Samples of fruit of the ‘Smoky’ cultivar (Figure 5A) subjected to ozone treatment, were found with significant increases in antioxidant potential on day 1 and day 3 of storage by 10.8% and 16.0%, respectively, compared to the untreated fruit. This increase is a result of the direct impact of ozone gas on the cell structures in the fruit. Piechowiak et al. [14] have shown that the enzyme system responsible for generating small-molecule antioxidants directly involved in the mitigation of effects produced by oxidative stress resulting from ozone exposure is activated in raspberries stored in an ozone atmosphere. As for saskatoon berries of the ‘Martin’ cultivar (Figure 5B), all the doses tested led to an increase in the content of vitamin C during storage. A similar effect was observed by Zhang et al. [22] in strawberries stored in ozone atmosphere, reflected by similar changes in vitamin C content.

#### 2.4.3. Ascorbic Acid Content

Gaseous ozone applied to raw plant materials is an abiotic agent that can induce oxidative stress in plant cells. However, the same gas applied under controlled conditions activates enzyme systems leading to the production of larger quantities of secondary metabolites, producing no destructive effect in the raw material exposed to ozone [12,15].

**Figure 6:**
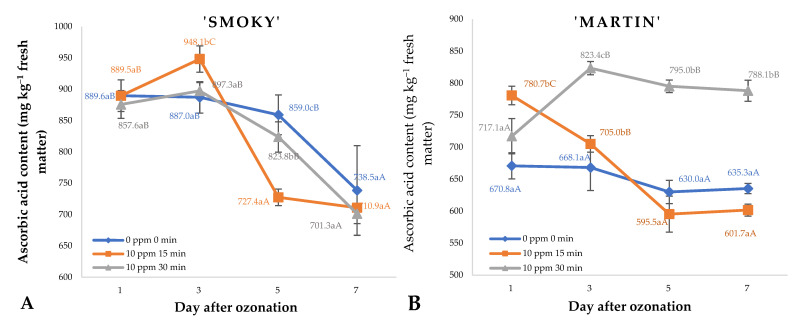
Ascorbic acid contents and standard error of the means in saskatoon berries of ‘Smoky’ (**A**) and ‘Martin’ (**B**) cultivars, relative to the dose of ozone (*n* = 3). Note: Differences in results between the dose of ozone on the specific days are indicated by different small letters and difference between assessment days are indicated by different capital letters. Significance level *p* < 0.05.

Effects of ozone treatment cyclically applied to saskatoon berries on the content of vitamin C during storage are shown in Figure 6. These effects were related to the saskatoon cultivar, the dose of ozone, and the duration of storage. Saskatoon berries of the ‘Smoky’ cultivar were found with the highest increase in vitamin C content on day 3 in the samples exposed to ozone at a rate of 10 ppm for 15 min. This is linked to the response of the fruit, which still exhibits the characteristics of living organisms by generating increased amounts of small-molecule antioxidants that may potentially mitigate the harmful effects of a powerful oxidant such as ozone. The decrease in vitamin C content observed in the subsequent days of storage was related to its natural decline associated with natural metabolic processes, which were slightly accelerated by the ozone treatment applied. Notably, however, on day 7 of storage, the contents of this compound did not differ significantly between the investigated experimental variants (Figure 6A). In the case of the ‘Martin’ cultivar, a similar trend in the changes of vitamin C content of saskatoon berries was observed in the control sample and in the fruit exposed to ozone at a rate of 10 ppm for 15 min. As for samples treated with the higher dose of ozone (10 ppm, 30 min), the contents of the compound fluctuated significantly, with the highest increase on day 3 and day 7 of storage (Figure 6B). Changes in the vitamin C content of fruit during storage in ozone atmosphere have also been reported for raspberries [23]. The fruit samples were stored in similar conditions, which resulted in a similar profile of changes in vitamin C contents during the storage.

## 3. Materials and Methods

### 3.1. Plant Material

The research material comprised samples of saskatoon berries (*Amelanchier alnifolia* Nutt.) of ‘Smoky’ and ‘Martin’ cultivars. The saskatoon berries of the relevant cultivars were collected at the stage of harvest maturity in the last week of June 2021. Mean water contents in the samples of the ‘Smoky’ and ‘Martin’ cultivars amounted to 88.9% ± 0.4, and 89.1% ± 0.3, respectively. The raw material designated for the study was characterised by colour, scent, and shape typical for fruit of this species. On the surface of the saskatoon fruits, there were no visible signs of pathogenic mould or yeast.

### 3.2. Ozone Treatment of the Plant Material

Immediately after they were harvested, saskatoon berry samples (1 kg) were treated with gaseous ozone. The ozone treatment was performed in compliance with the method described by Matłok et al. [15] in three independent replications. The procedure, aimed to improve the shelf life of saskatoon berries, applied ozone at a concentration of 10 ppm for 15 and 30 min. After that time, the source of ozone was disconnected. The treatment was repeated regularly every 24 h. The fruit samples were kept in food storage containers at 8 °C for 7 days. On days 1, 3, 5, and 7, samples of the fruit were collected to determine the water content, selected mechanical parameters (compressive and punching force), microbial stress, and the content of bioactive compounds (total polyphenols, vitamin C, and antioxidant potential) in the fruit. On the final day of the experiment, the samples were also assessed for the proportional content of healthy, mildew-infected, and rotten fruit relative to the conditions applied during the ozone treatment.

### 3.3. Measurement of Water Content

Measurements of water content in the saskatoon berries were conducted on the specific days of the experiment (day 1, 3, 5, and 7 following ozone treatment) using a laboratory drying oven SLW 115 (Pol-Eko-Aparatura, Wodzisław Śląski, Poland). The fruit samples were dried at a temperature of 105 °C to obtain dry matter.

### 3.4. Measurement of Mechanical Properties

#### 3.4.1. Indentation Test

On the specific days of storage, samples of *Amelanchier alnifolia* Nutt. Fruit of the ‘Smoky’ and ‘Martin’ cultivars were tested using an indenter with a diameter of φ = 2 mm. The measurements were performed using the testing machine Zwick/Roell Z010 (Zwick Roell Polska Sp. Z o.o. Sp. K., Wroclaw, Poland). The assessments were carried out in 36 replications for each sample (cultivar, conditions of ozone treatment process, storage duration). The resistance of the peel and pulp of saskatoon berries to the destructive force during the indentation test was determined under the following operating parameters: Fv = 0.05 N (initial force), V1 = 30 mm min^−1^ (crosshead return speed).

#### 3.4.2. Compression of Independent Specimens

Tests for the resistance of saskatoon berries to mechanical damage in a process of uniaxial compression were performed using a Zwick/Roell Z010 testing machine (Zwick Roell Polska Sp. Z o.o. Sp. K., Wroclaw, Poland). Measurements of compression force in ozone treated fruit and in untreated specimens (control) of the cultivars investigated were carried out on days 1, 3, 5, and 7 of storage. The assessments were performed in 36 replications for each experimental variant and the following operating conditions: initial force for specimen tension F = 2 N, and speed of the module during compression test 0.5 mm s^− 1^. Saskatoon berry samples designated for the mechanical tests comprised fruit of the specific experimental variant with no visible signs of infection with diseases occurring during storage.

### 3.5. Microbial Load

Saskatoon berries representing the specific experimental variants were subjected to microbiological testing on days 1, 3, 5, and 7 in storage (following ozone treatment). The counts of mesophilic lactic acid bacteria, aerobic bacteria, yeast, and mould were determined in accordance with the methodology described by Matłok et al. [18].

### 3.6. Content of Bioactive Compounds

Samples of saskatoon berries designated for chemical assays were prepared in accordance with the method described by Matłok et al. [18]. The obtained supernatants were then subjected to assays in order to determine the total content of polyphenols in line with the methodology proposed by Matłok et al. [24] and total antioxidant potential following the CUPRAC method described by Matłok et al. [25].

### 3.7. Total Ascorbic Acid Assay

Samples of saskatoon berries (5 g) were homogenized with 50 mL of 2% oxalic acid and centrifuged at 10,000× *g* for 30 min. The supernatant was subjected to ascorbic acid analysis using the titrimetric method with 2,6- dichlorophenolindophenol. Briefly, 10 mL of the extract was immediately titrated with 2,6-dichlorophenolindophenol (Sigma-Aldrich, Steinheim, Germany) at the concentration 25 mg in 100 mL [23]. Vitamin C content was calculated based on a calibration curve prepared by titration of a series standards of ascorbic acid at the concentrations of 0.1, 0.25, 0.5, and 1 mg mL^−1^.

### 3.8. Statistical Analysis

Multidimensional analysis of variance (ANOVA) of results was conducted at the significance level α = 0.05 utilising STATISTICA 13.1 software (TIBCO Software Inc., Hillview Avenue, Palo Alto, CA, USA). The mean values calculated from the three independent replications were analysed statistically by comparing the results between the variants of the experiment.

## 4. Conclusions

Ozone treatment applied to saskatoon berries significantly affected the quality and shelf life of the fruit. The effect varied considerably relative to the cultivar, conditions of ozone treatment, and duration of storage. The findings show that the content of bioactive compounds increased on the initial days of storage, and the effect was related to the dose of gaseous ozone applied. The highest increase, compared to the control sample (untreated fruit), was found 24 h after ozone treatment (10 ppm 30 min). It was observed that decay of bioactive compounds and growth of micro-organisms on the surface of fruit were inhibited while kept in storage, irrespective of the conditions of the ozone treatment. This resulted in a decreased loss of water content and lower incidence of diseases occurring in storage, which directly led to significantly reduced storage losses during the relevant period.

## Figures and Tables

**Table 1:** Microbial load in saskatoon berries on the first, third, fifth, and seventh day after ozonation relative to the duration of the treatment (*n* = 3).

Date of Measurement	Daily Dose of Ozone		Count of Aerobic Bacteria (log cfu g^−1^)		Count of Yeast and Mould (log cfu g^−1^)	
		Cultivar	‘Matrin’	‘Smoky’	‘Matrin’	‘Smoky’
1 day of ozonation	0 ppm 0 min		6.20 ^aA^	5.40 ^aA^	3.93 ^aA^	3.74 ^aA^
	10 ppm 15 min		6.14 ^aA^	5.84 ^aA^	3.58 ^aA^	3.11^a A^
	10 ppm 30 min		5.46 ^bA^	5.72 ^aB^	3.36 ^aA^	3.00 ^bA^
3 days of ozonation	0 ppm 0 min		6.49 ^aA^	5.96 ^aA^	3.74 ^aA^	3.88 ^aA^
	10 ppm 15 min		6.43 ^aA^	5.81 ^aA^	3.66 ^aA^	3.76 ^aA^
	10 ppm 30 min		6.28 ^aB^	5.99 ^aB^	3.49 ^aA^	3.66 ^aA^
5 days of ozonation	0 ppm 0 min		6.26 ^aA^	5.41 ^aA^	3.89 ^aA^	3.93 ^aA^
	10 ppm 15 min		6.08 ^aA^	5.11 ^aA^	3.72 ^aA^	3.92 ^aA^
	10 ppm 30 min		6.06 ^aB^	5.17 ^aA^	3.56 ^aA^	3.60 ^aA^
7 days of ozonation	0 ppm 0 min		6.32 ^aA^	5.32 ^aA^	3.91 ^aA^	3.97 ^aA^
	10 ppm 15 min		6.32 ^aA^	5.34 ^aA^	3.53 ^aA^	3.92 ^aA^
	10 ppm 30 min		6.26 ^aB^	5.00 ^aA^	3.45 ^aA^	3.59 ^aA^

Note: Differences in results between the dose of ozone on specific days are indicated by different small letters, and differences between assessment days are indicated by different capital letters. Significance level *p* < 0.05.

## Data Availability

The data presented in this study are available in this article.

## References

[1] Lachowicz S., Oszmiański J., Pluta S. (2017). The composition of bioactive compounds and antioxidant activity of Saskatoon berry (*Amelanchier alnifolia* Nutt.) genotypes grown in central Poland. Food Chem..

[2] Mazza G., Cattrell T. (2008). Carotenoids and cyanogenic glucosides in Saskatoon berries (*Amelanchier alnifolia* Nutt.). J. Food Compos. Anal..

[3] Ribeiro de Souza D., Willems J.L., Low N.H. (2019). Phenolic composition and antioxidant activities of saskatoon berry fruit and pomace. Food Chem..

[4] Lachowicz S., Seliga Ł., Pluta S. (2020). Distribution of phytochemicals and antioxidative potency in fruit peel, flesh, and seeds of Saskatoon berry. Food Chem..

[5] Ozga J.A., Saeed A., Reinecke D.M. (2006). Anthocyanins and nutrient components of saskatoon fruits (*Amelanchier alnifolia* Nutt.). Can. J. Plant Sci..

[6] Juríková T., Balla S., Sochor J., Pohanka M., Mlcek J., Baron M. (2013). Flavonoid Profile of Saskatoon Berries (*Amelanchier alnifolia* Nutt.) and Their Health Promoting Effects. Molecules.

[7] Mitra P., Meda V., Green R. (2013). Effect of dryling techniques on the retention of antioxidant activities of Saskatoon berries. Int. J. Food Stud..

[8] Zhao R., Khafipour E., Sepehri S., Huang F., Beta T., Shen G.X. (2019). Impact of Saskatoon berry powder on insulin resistance and relationship with intesinal microbiota in high fat-high sucrose diet-induce dobese mice. J. Nutr. Biochem..

[9] Moyo M., Aremu A.O., Plačková L., Plíhalová L., Pĕnčík A., Novák O., Holub J., Doležal K., Van Staden J. (2018). Deciphering the growth pathern and phytohormonal content in Saskatoon berry (*Amelanchier alnifolia* Nutt.) in response to in vitro cytokinin application. New Biotechnol..

[10] Zapałowska A., Matłok N., Zardzewiały M., Piechowiak T., Balawejder M. (2021). Effect of Ozone Treatment on the Quality of Sea Buckthorn (*Hippophae rhamnoides* L.). Plants.

[11] Mashabela M., Mahajan P.V., Sivakumar D. (2019). Influence of different types of modified atmosphere packaging films and storage time on quality and bioactive compounds in fresh-cut cauliflower. Food Packag. Shelf Life.

[12] Zardzewiały M., Matlok N., Piechowiak T., Gorzelany J., Balawejder M. (2020). Ozone Treatment as a Process of Quality Improvement Method of Rhubarb (*Rheum rhaponticum* L.) Petioles during Storage. Appl. Sci..

[13] Antos P., Piechowicz B., Gorzelany J., Matłok N., Migut D., Józefczyk R., Balawejder M. (2018). Effect of Ozone on Fruit Quality and Fungicide Residue Degradation in Apples during Cold Storage. Ozone Sci. Eng..

[14] Piechowiak T., Balawejder M. (2019). Impact of ozonation process on the level of selected oxidative stress markers in raspberries stored at room temperature. Food Chem..

[15] Matlok N., Piechowiak T., Gorzelany J., Zardzewiały M., Balawejder M. (2020). Effect of Ozone Fumigation on Physiological Processes and Bioactive Compounds of Red-Veined Sorrel (*Rumex sanguineus* ssp. *sanguineus*). Agronomy.

[16] Matłok N., Gorzelany J., Piechowiak T., Antos P., Zardzewiały M., Balawejder M. (2020). Impact of Ozonation Process on the Content of Bioactive Compounds with Antioxidant Properties in Scots Pine (*Pinus sylvestris* L.) Shoots as Well as Yield and Composition of Essential Oils. Acta Univ. Cibiniensis Ser. E Food Technol..

[17] Gorzelany J., Migut D., Matłok N., Antos P., Kuźniar P., Balawejder M. (2017). Impact of Pre-Ozonation on Mechanical Properties of Selected Genotypes of Cucumber Fruits During the Souring Process. Ozone Sci. Eng..

[18] Matłok N., Piechowiak T., Zardzewiały M., Gorzelany J., Balawejder M. (2020). Effects of Ozone Treatment on Microbial Status and the Contents of Selected Bioactive Compounds in *Origanum majorana* L. Plants. Plants.

[19] Piechowiak T., Antos P., Józefczyk R., Kosowski P., Skrobacz K., Balawejder M. (2019). Impact of Ozonation Process on the Microbiological Contamination and Antioxidant Capacity of Highbush Blueberry (*Vaccinum corymbosum* L.) Fruit during Cold Storage. Ozone Sci. Eng..

[20] O’Donnell C., Tiwari B.K., Cullen P.J., Rice R.G. (2012). Ozone in Food Processing.

[21] Galazka A. (2013). Conversion of phenolic compounds and the role of L-phenylalanine amonia lyase (PAL) in the induction of plant defense mechanisms. Pol. J. Agron..

[22] Zhang X., Zhang Z., Wang L., Zhang Z., Jing L., Zhang C. (2011). Impact of ozone on quality of strawberry during cold storage. Front. Agric. China.

[23] Piechowiak T., Antos P., Kosowski P., Skrobacz K., Józefczyk R., Balawejder M. (2019). Impact of ozonation process on the microbiological and antioxidant status of raspberry (*Rubus ideaeus* L.) fruit during storage at room temperature. Agric. Food Sci..

[24] Matłok N., Stępień A.E., Gorzelany J., Wojnarowska-Nowak R., Balawejder M. (2020). Effects of Organic and Mineral Fertilization on Yield and Selected Quality Parameters for Dried Herbs of Two Varieties of Oregano (*Origanum vulgare* L.). Appl. Sci..

[25] Matłok N., Kapusta I., Piechowiak T., Zardzewiały M., Gorzelany J., Balawejder M. (2021). Characterisation of Some Phytochemicals Extracted from Black Elder (*Sambucus nigra* L.) Flowers Subjected to Ozone Treatment. Molecules.

